# From biomarker to targeted therapy: investigating trophoblast cell-surface antigen 2 expression in triple-negative breast cancer– insights from the national cancer institute

**DOI:** 10.1186/s12885-025-14402-7

**Published:** 2025-06-05

**Authors:** Noura A. A. Ebrahim, Mustafa A. Hussein, Mohamed Emam Sobeih, Nancy H. Amin

**Affiliations:** 1 Oncologic Pathology Department, National Cancer Institute (NCI) - Cairo University, Giza, Egypt; 2 Medical Oncology Department, National Cancer Institute (NCI) - Cairo University, Giza, Egypt

**Keywords:** TROP-2, Triple-negative breast cancer, Immunohistochemistry, Precision oncology, Prognostic biomarker

## Abstract

**Background:**

Triple-negative breast cancer (TNBC) is characterized by its aggressive behavior and limited treatment options, primarily due to the lack of expression of estrogen receptor (ER), progesterone receptor (PR), and human epidermal growth factor receptor 2 (HER2). TROP-2, a transmembrane glycoprotein, exhibits notable overexpression in a spectrum of highly aggressive cancers including pancreatic, gastric, and ovarian cancers. This overexpression has established TROP-2 as a key prognostic biomarker and a promising target for therapeutic intervention.

**Objective:**

This research examines the correlation between TROP-2 expression and clinicopathological features in TNBC, assessing its utility as both a prognostic indicator and a candidate for targeted precision therapy.

**Methods:**

Retrospective analysis of 80 TNBC patient samples from January 2016 to December 2019 at the National Cancer Institute, Cairo University, Egypt was carried out. Formalin-fixed, paraffin-embedded (FFPE) tissues were evaluated for TROP-2 expression using immunohistochemistry. Clinical and pathological data including patient demographics, tumor characteristics, treatment modalities, and survival outcomes were gathered. TROP-2 expression was correlated with clinicopathological variables and survival metrics (overall survival, OS; disease-free survival, DFS).

**Results:**

A high expression of TROP-2 was observed in 78% of cases, exhibiting notable heterogeneity in intensity, proportion of positive cells, and H-score. TROP-2 expression correlated with larger tumor dimensions and advanced nodal involvement, suggesting its contribution to tumor aggressiveness. Elevated TROP-2 intensity and H-scores were significantly associated with poorer overall survival (OS; *p* = 0.003 and *p* = 0.007, respectively) and disease-free survival (DFS; *p* = 0.002 for both). Multivariate analysis revealed TROP-2 intensity, percentage of expression, and H-score as independent predictors of OS (*p* = 0.02, 0.001, and 0.012, respectively). Similarly, these variables were identified as independent prognostic indicators for DFS, with significant p-values of 0.002, 0.009, and 0.002.

**Conclusions:**

Our research validates TROP-2 overexpression as an essential prognostic marker and a potential therapeutic target in TNBC. The results endorse the use of TROP-2 expression levels for patient categorization, thereby advancing the implementation of personalized treatment strategies and accelerating the progression towards precision oncology.

## Keypoints

This research explores the role of TROP-2, a transmembrane glycoprotein that is markedly overexpressed in triple-negative breast cancer (TNBC), a subtype characterized by its aggressive nature and poor prognosis. Through the analysis of 80 TNBC cases, the study reveals a significant association between high TROP-2 expression and unfavorable clinicopathological parameters, such as increased tumor size, advanced nodal involvement, and reduced overall and disease-free survival. These findings underscore the potential of TROP-2 as a critical prognostic biomarker and a viable therapeutic target, with emerging strategies like the antibody-drug conjugate sacituzumab govitecan offering new avenues for personalized treatment in TNBC.

## Introduction

Cancer encompasses a diverse group of diseases characterized by abnormal cell proliferation, invasion of surrounding tissues, and the potential to metastasize to distant organs. Due to its complex pathophysiology, effective cancer management necessitates a comprehensive diagnostic and therapeutic approach tailored to the molecular and clinical features of each cancer subtype [1–3].

Among breast cancer subtypes, triple-negative breast cancer (TNBC) is distinguished by its aggressive nature and limited treatment options. Lacking expression of estrogen receptor (ER), progesterone receptor (PR), and human epidermal growth factor receptor 2 (HER2), TNBC is unresponsive to endocrine and HER2-targeted therapies, making it particularly challenging to treat. It accounts for approximately 15–20% of breast cancer cases globally and poses a significant burden due to its poor prognosis and high recurrence rate [4–6]. Despite advances in therapy, TNBC is frequently associated with early metastasis and unfavorable clinical outcomes, highlighting the urgent need for novel treatment modalities [7, 8].

Trophoblast cell surface antigen 2 (TROP-2), a transmembrane glycoprotein encoded by the *TACSTD2* gene, has emerged as a promising therapeutic target in TNBC. Normally involved in cellular adhesion and signaling, TROP-2 becomes pathologically upregulated in several cancers, including TNBC, where it contributes to tumor progression, metastasis, and therapy resistance [9]. Notably, TROP-2 overexpression is observed in up to 80% of TNBC cases and has been correlated with enhanced tumor aggressiveness and diminished survival, underlining its potential as both a biomarker and a target for intervention [10–12].

Structurally, TROP-2 is a type I transmembrane protein comprising an extracellular domain, a transmembrane region, and a cytoplasmic tail containing a phosphatidylinositol (PIP2)-binding motif. This motif mediates the activation of key signaling cascades such as ERK/MAPK, PI3K/AKT, and NF-κB, all of which regulate critical processes like proliferation, apoptosis resistance, and epithelial-to-mesenchymal transition (EMT)—pathways frequently hijacked in cancer [13, 14].

As an oncogenic regulator, TROP-2 promotes malignancy in various epithelial cancers, including TNBC, by facilitating calcium-dependent signaling pathways that enhance cell proliferation, invasion, and survival. Its overexpression in TNBC appears to compensate for the loss of ER, PR, and HER2-mediated signaling by sustaining oncogenic pathways that fuel tumor growth and resistance to therapy [10, 15–17]. In this context, TROP-2 is not only a driver of tumor aggressiveness but also a viable target for precision oncology [18–20].

At the mechanistic level, TROP-2 facilitates EMT by interacting with β-catenin and claudin complexes, disrupting adherens junctions and promoting nuclear translocation of β-catenin. This, in turn, activates EMT transcription factors such as Snail, Slug, and Twist, enhancing cell motility and metastatic potential [21–24]. In mesenchymal-like tumor cells, TROP-2 sustains oncogenic signaling through PI3K/AKT and MAPK pathways independently of traditional hormone receptors, promoting survival, angiogenesis, and immune evasion [25–27].

The advent of TROP-2-targeted therapies, especially antibody-drug conjugates (ADCs) like sacituzumab govitecan, marks a pivotal advancement in the treatment of triple-negative breast cancer (TNBC). Sacituzumab govitecan is designed to selectively deliver the potent cytotoxic agent SN-38 to tumor cells exhibiting high TROP-2 expression. By exploiting the abundant surface localization of TROP-2 in TNBC cells, this therapeutic strategy enables precise intracellular delivery of the drug, thereby enhancing antitumor efficacy while minimizing damage to surrounding healthy tissues [12]. Beyond its cytotoxic activity, this approach also disrupts key oncogenic processes, notably epithelial-to-mesenchymal transition (EMT) and associated pro-survival signaling pathways, which are often responsible for therapeutic resistance in receptor-negative breast cancers [11, 28–30].

TROP-2 has emerged as a clinically significant biomarker in triple-negative breast cancer (TNBC), owing to its multifaceted role in both epithelial and mesenchymal tumor compartments. Under normal physiological conditions, TROP-2 contributes to maintaining cell adhesion and tissue architecture, particularly within trophoblastic tissues. However, in the malignant setting of TNBC, its aberrant overexpression disrupts these functions, driving enhanced cellular proliferation, invasion, and resistance to apoptosis [9, 31–36]. This pathological overexpression is closely associated with the suppression of E-cadherin, a key adhesion molecule, thereby promoting epithelial-to-mesenchymal transition (EMT) and fostering a more invasive and aggressive tumor phenotype [37].

Elevated TROP-2 expression correlates with unfavorable prognostic indicators, including reduced overall and disease-free survival and an increased likelihood of distant metastasis. Multiple studies have identified TROP-2 as an independent predictor of poor clinical outcomes in TNBC, underscoring its prognostic utility [38, 39]. Experimental data further support its central role in driving EMT and tumor aggressiveness, highlighting its potential not only as a biomarker for risk stratification but also as a key target in developing more effective therapeutic strategies [40].

Beyond prognosis, TROP-2 has emerged as a predictive marker for therapeutic response. Patients with high TROP-2 expression show greater benefit from TROP-2-directed ADCs, reflecting the value of stratifying patients based on biomarker expression [41]. Among these agents, sacituzumab govitecan remains the most advanced, combining a TROP-2-specific antibody with SN-38 to ensure targeted cytotoxicity. The ASCENT trial notably demonstrated significant improvements in survival outcomes, establishing this ADC as a viable option for advanced TNBC [42].

Emerging therapies are also exploring novel TROP-2-targeted strategies, including bispecific antibodies that simultaneously engage TROP-2 and immune checkpoints such as PD-L1, aiming to enhance antitumor immunity. Additionally, small-molecule inhibitors targeting TROP-2-mediated signaling are under development, broadening the therapeutic arsenal available for managing TROP-2-expressing tumors [43–46].

The present study aims to investigate the clinicopathological implications of TROP-2 expression in TNBC and assess its utility as both a prognostic indicator and a therapeutic target. By correlating TROP-2 levels with tumor characteristics and clinical outcomes, this research seeks to deepen our understanding of its role in TNBC pathobiology and treatment.

## Materials and methods

### Study design and sample selection

This study was designed to assess tissue samples from patients diagnosed with triple-negative breast cancer (TNBC) over a four-year timeframe, from January 2016 to December 2019, at our institution. Ethical approval was obtained from the Institutional Review Board of the National Cancer Institute, Cairo University, ensuring that all procedures adhered to established ethical standards.

Archived formalin-fixed, paraffin-embedded (FFPE) tissue blocks were retrieved from the pathology department’s records. Cases were selected based on a confirmed diagnosis of TNBC, which was verified by reviewing archived immunohistochemical slides for estrogen receptor (ER), progesterone receptor (PR), and HER2 expression. All tumors in the cohort demonstrated complete negativity for ER, PR, and HER2 expression. None of the cases met the criteria for HER2-low classification, thereby affirming strict compliance with the diagnostic criteria for TNBC. To maintain the quality and robustness of the study, we excluded samples with incomplete clinical data or insufficient tissue for further analysis. Ultimately, 80 cases that fulfilled the inclusion criteria were incorporated into the study.

### Clinicopathologic data collection

For each patient, we systematically collected detailed clinical and pathological data to construct a comprehensive dataset. The following parameters were documented:

- Patient age
- Tumor laterality (right, left or bilateral)
- Tumor localization within the breast
- Surgical procedure performed (conservative breast surgery or modified radical mastectomy)
- Tumor size, expressed in centimeters
- Histopathological tumor classification (e.g., invasive ductal carcinoma)
- Presence of ductal carcinoma in situ (DCIS)
- Lymph node status (positive or negative), (if applicable)
- Evidence of capsular invasion (if applicable)
- Presence of lymphovascular emboli
- TNM staging, including:
- T stage (largest tumor diameter)
- N stage (extent of lymph node involvement), (if applicable)
- Chemotherapy regimen (neoadjuvant, adjuvant therapy or combined)
- Radiotherapy details, specifying whether radiation was administered as neoadjuvant treatment, adjuvant therapy or combined
- Recurrence data, including both local and metastatic recurrence
- Death status (alive or deceased).

### Immunohistochemistry procedure

To classify triple-negative breast cancer (TNBC) into molecular subtypes, immunohistochemical (IHC) analysis was conducted using markers for epidermal growth factor receptor (EGFR), cytokeratin 5/6 (CK5/6), and androgen receptor (AR). Based on these immunoprofiles, tumors were categorized into three distinct subtypes according to established criteria: Basal-like (BL), Mesenchymal (M), and Luminal Androgen Receptor (LAR). Specifically, tumors were labeled as Basal-like if they expressed EGFR and/or CK5/6 while lacking AR expression, Mesenchymal if all three markers were negative, and Luminal AR if AR was positive, regardless of EGFR and CK5/6 status, following validated immunohistochemical algorithms aligned with transcriptomic classifications [47]. For this purpose, monoclonal antibodies targeting EGFR (clone 31G7, Invitrogen, USA; 1:50 dilution), CK5/6 (clone D5/16B4, Dako, Denmark; 1:50 dilution), and AR (clone SP107, Abcam, UK; 1:100 dilution) were employed, all optimized for the Ventana BenchMark ULTRA automated staining platform. The OptiView DAB IHC Detection Kit (Ventana) was used for detection, with positive and negative controls included in each run. Additionally, TROP-2 expression was assessed using a monoclonal TROP-2 antibody (clone B9, Santa Cruz Biotechnology) validated for the same platform, with placental tissue as a positive control and negative controls achieved by omitting the primary antibody. Four-micron FFPE tissue sections underwent deparaffinization, heat-induced antigen retrieval with Ventana’s CC1 buffer, and incubation with SP295 and TROP-2 B9 antibodies, followed by visualization using the OptiView DAB detection system and hematoxylin counterstaining. TROP-2 positivity was defined as membranous staining, with or without cytoplasmic staining, in at least 10% of tumor cells. Staining was evaluated for both intensity (graded 0 to 3+) and the percentage of positive tumor cells (≤ 50% or > 50%), and an H-score was calculated by multiplying the intensity score by the percentage of tumor cells at each level of staining, yielding a total score ranging from 0 to 300 to provide a quantitative assessment of expression [16]. Three independent pathologists, blinded to clinical outcomes, reviewed the slides, and any scoring discrepancies were resolved through consensus.

### Statistical analysis

The data were systematically analyzed to investigate the associations between TROP-2 expression and clinicopathological characteristics, recurrence patterns, and survival outcomes. Survival analyses, encompassing both overall survival (OS) and disease-free survival (DFS), were performed using R programming (version 4.4.2) and SPSS (version 27). OS is defined as the time from diagnosis to death from any cause, while DFS refers to the time from diagnosis to the first occurrence of recurrence or metastasis. A p-value of less than 0.05 was considered indicative of statistical significance.

## Results

**Fig. 1:**
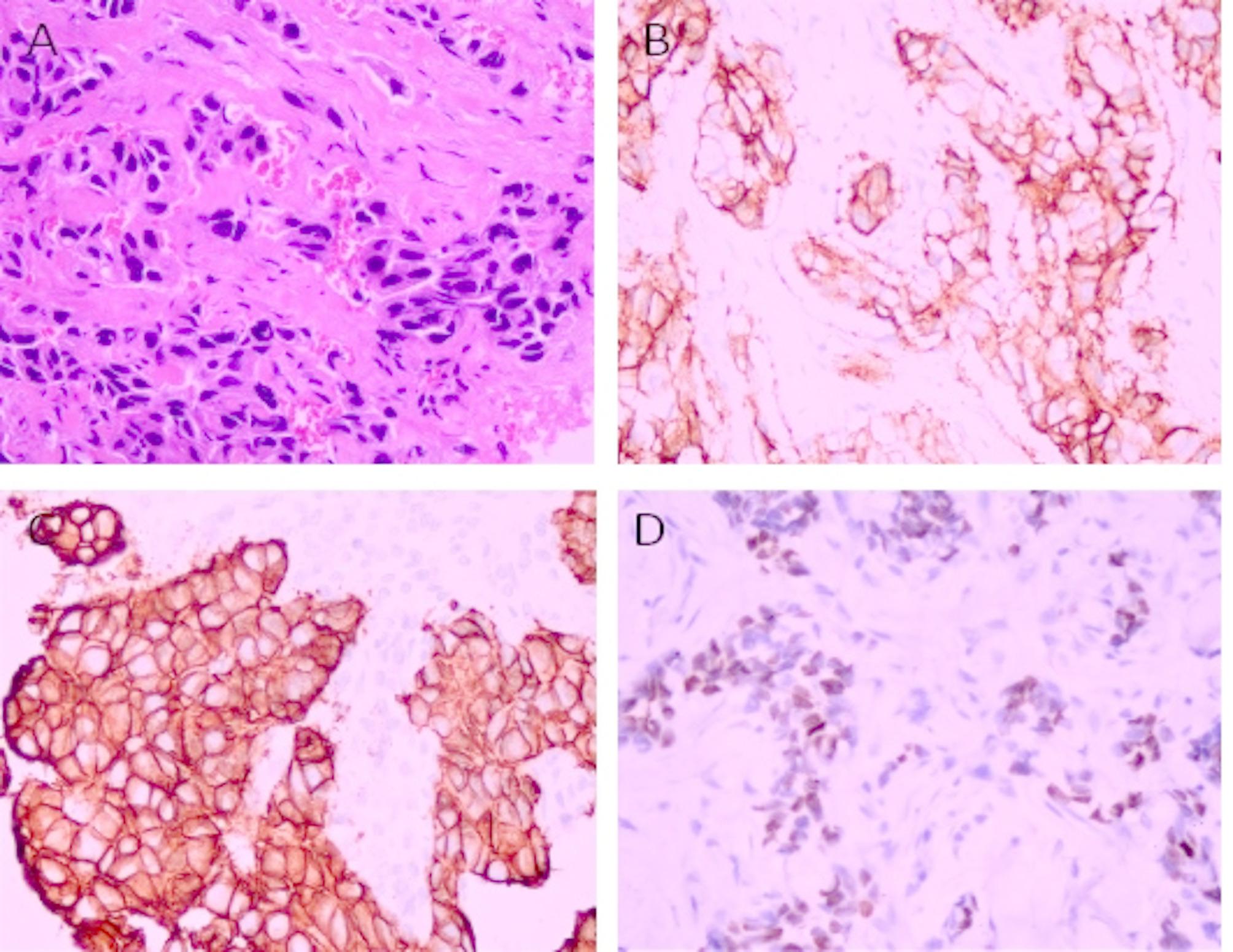
Histopathological and immunohistochemical characterization of invasive ductal carcinoma, grade 3, illustrating features of molecular subtyping in triple-negative breast cancer (TNBC). **(A)** Hematoxylin and eosin (H&E) stained section reveals prominent nuclear pleomorphism with minimal glandular differentiation, consistent with high-grade morphology (original magnification ×400). **(B)** Immunohistochemical staining for epidermal growth factor receptor (EGFR) demonstrates a strong membranous positivity (×400), supporting basal-like features. **(C)** Cytokeratin 5/6 (CK5/6) immunostaining shows diffuse cytoplasmic and membranous positivity (×400), confirming basal-like differentiation. **(D)** Androgen receptor (AR) immunostaining highlights distinct nuclear positivity (×400), indicative of the luminal androgen receptor (LAR) subtype

The follow-up period ranged from 10 to 104 months, with a mean of 90.7 (± 3.15 SE), Patient ages spanned 25 to 84 years, with a median age of 53.5 years. Tumor sizes varied between 1 cm and 8 cm, with a median size of 3 cm. Overall survival rate is 80.7% (95% CI: 72.4%; 90.0%), and disease free survival rate is 71.0% (**95% CI**: 60.7%; 83.0%). Recurrence was observed in 21 patients, with 5 experiencing local recurrence, 17 exhibiting metastatic recurrence, and 1 patient presenting both local and metastatic recurrence. All cases were grade 3 tumors and the majority of them were diagnosed as invasive duct carcinoma grade 3 (68 cases, 85%, Fig. 1).

### Distribution of TROP2 expression across tumor samples

The assessment of TROP2 expression revealed distinct patterns across intensity levels, percentage expression, and H-scores.

- Intensity: The distribution of TROP2 intensity levels was as follows: strong (3+) in 33 patients (41%), moderate (2+) in 36 patients (45%), and weak (1+) in 11 patients (14%).
- Percentage Expression: High expression (greater than 50%) was observed in 61 patients (78%), while 19 patients (22%) exhibited low expression (50% or less).
- H-Score: In the cohort of 80 patients, 25 patients (31%) exhibited high H-scores (ranging from 200 to 300), 37 patients (46%) had moderate scores (ranging from 100 to 199), and 18 patients (23%) had low scores (less than 100).

These results highlight the heterogeneous nature of TROP2 expression, prompting further investigation into its prognostic significance.

### Associations between TROP2 expression and clinicopathological features

#### Tumor size

- Higher TROP2 intensity, and extensive expression were strongly associated with larger tumor sizes (≥ 3 cm) (*p* = 0.006).

#### Nodal status

**Fig. 2:**
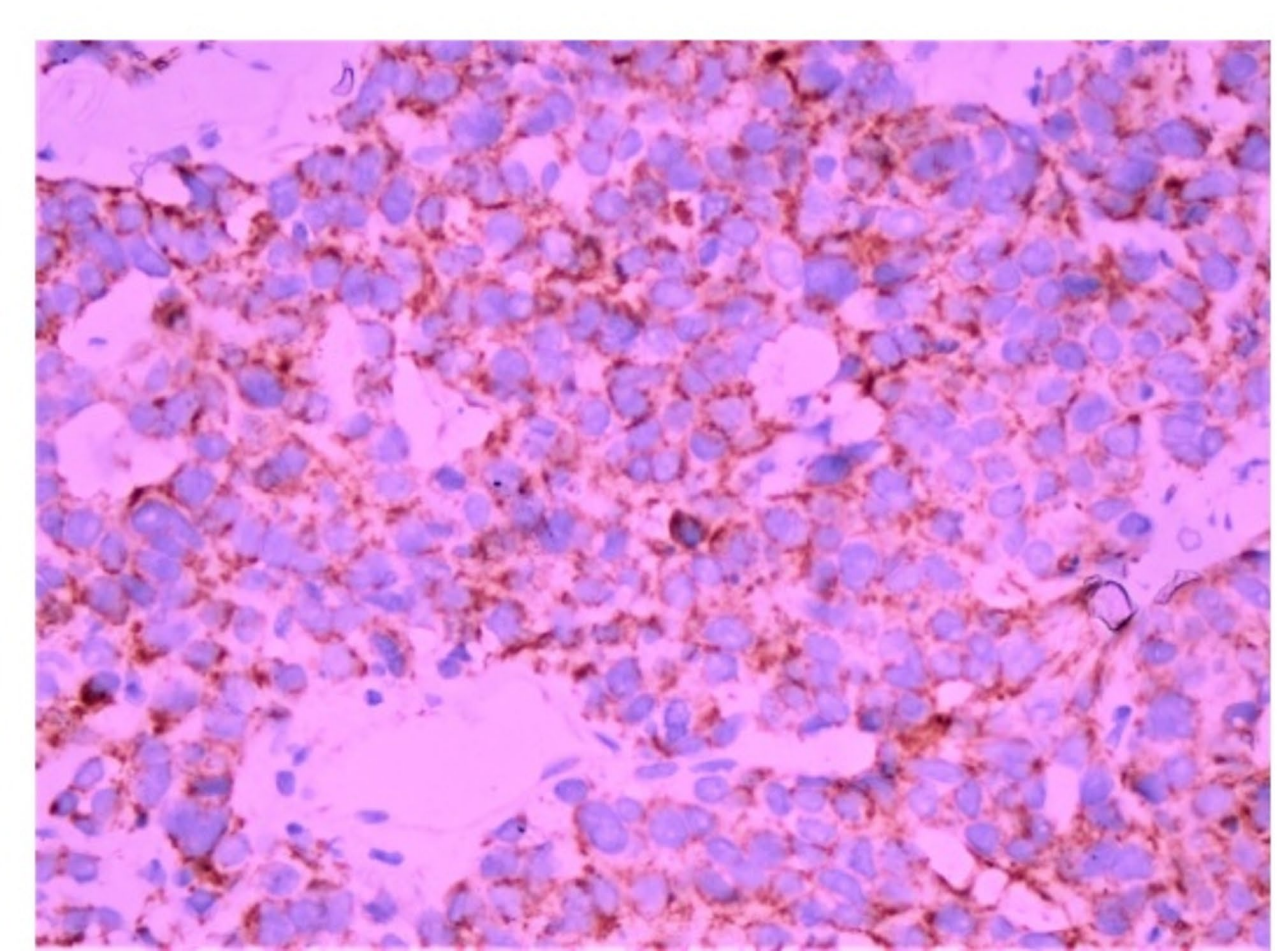
Immunohistochemical staining for TROP2 showing a score of 1+ (original magnification ×400). DAB was utilized as the chromogen for detection and visualization

**Fig. 3:**
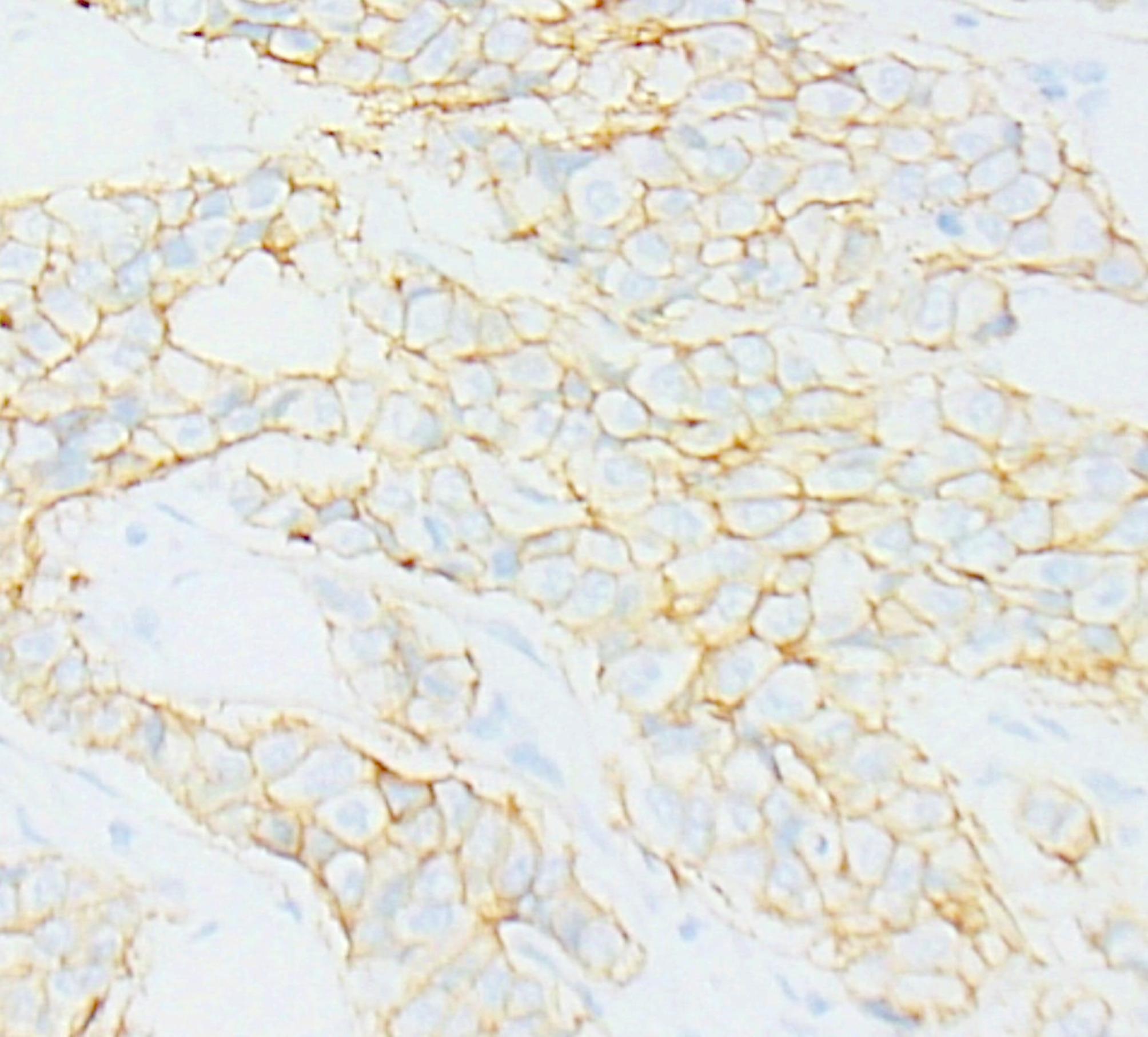
Immunohistochemical staining for TROP2 showing a score of 2+ (original magnification ×400). DAB was utilized as the chromogen for detection and visualization

**Fig. 4:**
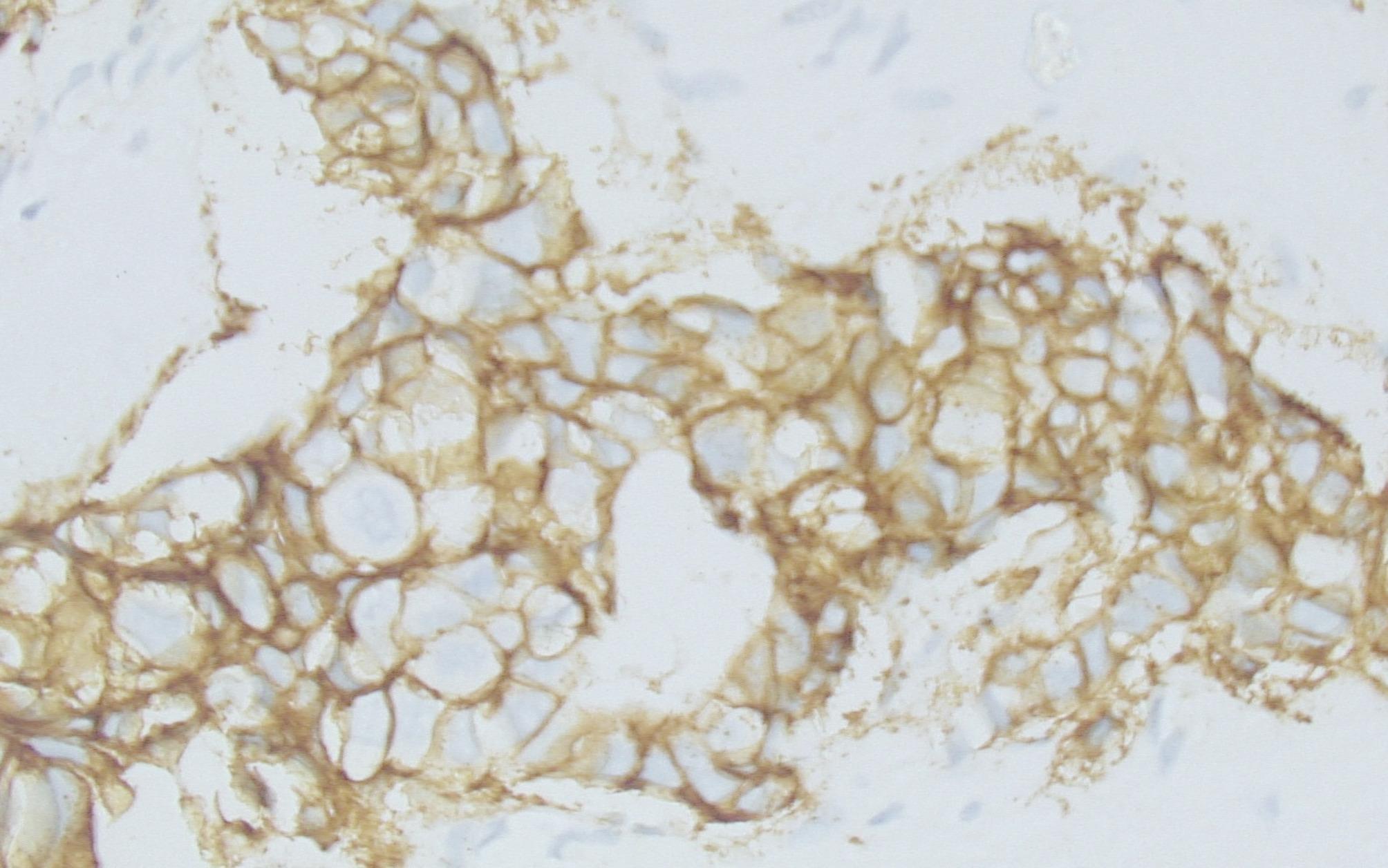
Immunohistochemical staining for TROP2 showing a score of 3+ (original magnification ×400). DAB was utilized as the chromogen for detection and visualization

- Advanced nodal involvement correlated significantly with TROP2 positivity (p value = 0.002 in the setting of TROP2 intensity (Figs. 2, 3 and 4), 0.0002 in the setting of TROP2 extent of expression, and 0.013 in the setting of correlation with TROP2 H score).

A detailed summary of the initial clinical parameters of the study population is presented in Table 1. Table 2 outlines the associations between TROP2 expression—assessed through H-score, percentage of positive tumor cells, and staining intensity—and key clinicopathological variables. In Table 3, a significant relationship between TROP2 expression and molecular subtypes is demonstrated. Notably, tumors classified under the basal-like (BL) subtype exhibited substantially elevated H-scores, higher percentages of TROP2-positive cells, and stronger staining intensities when compared to the luminal androgen receptor (LAR) and mesenchymal (M) subtypes (*p* < 0.001 for both H-score and intensity; *p* = 0.008 for percentage expression), suggesting a distinct expression profile of TROP2 among different molecular classes. Table 4 summarizes the therapeutic strategies applied within the cohort. The majority of patients received adjuvant chemotherapy (88.8%) and adjuvant radiotherapy (67.5%), whereas only a few underwent neoadjuvant therapy or were managed with palliative intent. Due to the limited number of patients receiving neoadjuvant treatment (*n* = 2), pathological assessment of treatment response was not feasible. Nevertheless, recurrence-related parameters were evaluated as indirect indicators of treatment efficacy, as outlined below.

### Prognostic implications of TROP2 expression metrics

#### Overall survival (OS)

**Fig. 5:**
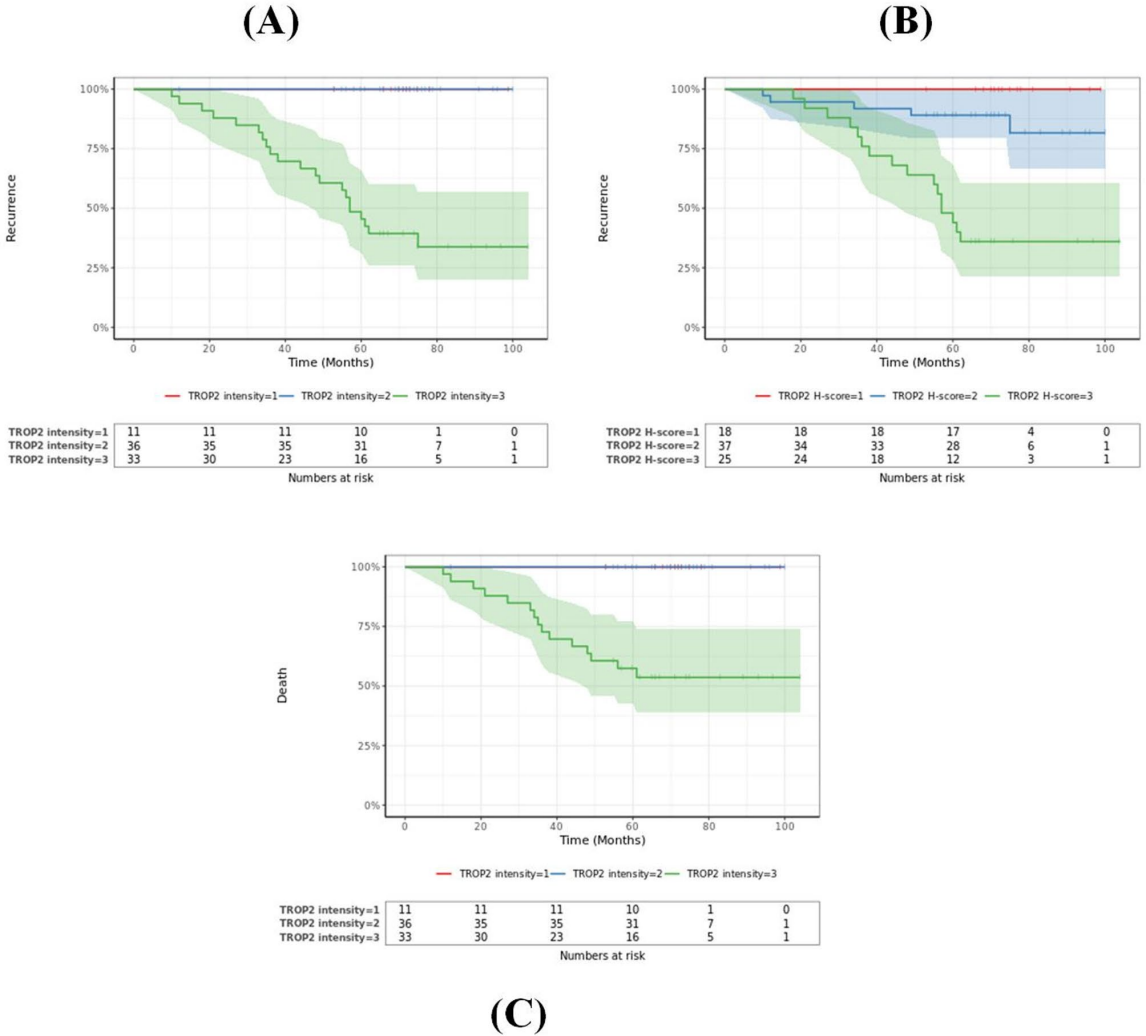
Illustrates recurrence in relation to TROP2 intensity and TROP2 H-score, (**A**& **B** respectively). **C** illustrates Death frequency in relation to TROP2 intensity

**Fig. 6:**
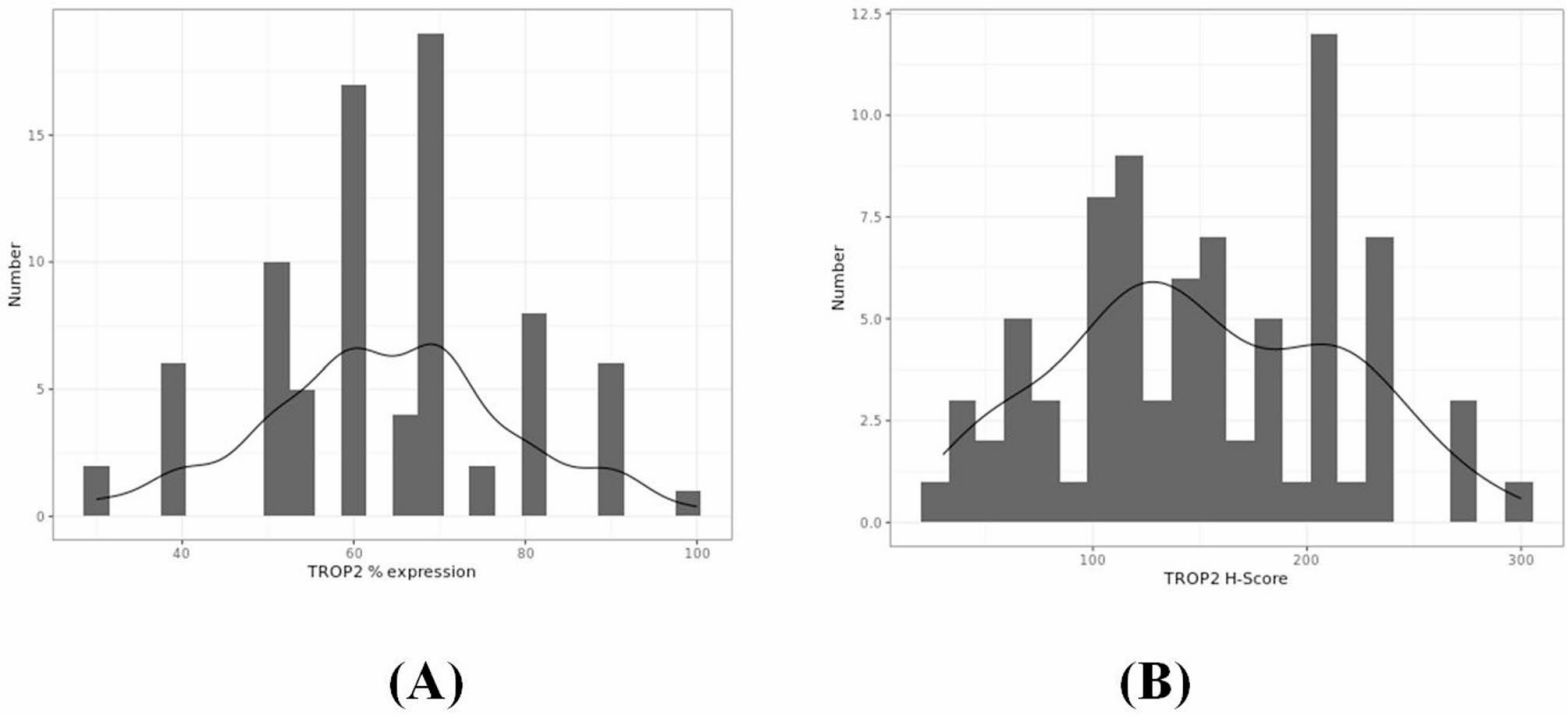
(**A**): Illustrate TROP2% expression (x axis) in relation to number of deaths (y axis), minimum = 30, maximum = 100, with a median **[Q25-75]** of 62.5 [55.0; 70.0], **mean (sd)**: 63.9 (14.6). (**B**): Illustrate TROP2 H-score (x axis) in relation to number of deaths (y axis), minimum = 30, maximum = 300, with a median **[Q25-75]** of 140 [108; 210], **mean (sd)**: 151 (65.0)

The analysis of TROP2 expression parameters demonstrated significant correlations with overall survival outcomes, (Figs. 5 and 6):

TROP2 Intensity:

- Kaplan-Meier survival analysis revealed that patients exhibiting strong TROP2 intensity (3+) had significantly shorter OS compared to those with moderate and weak intensity (2 + and 1 + respectively).
- This finding was statistically robust (p = < **0.001**), highlighting TROP2 intensity as a pivotal prognostic factor for survival.

#### TROP2 H-score

- Patients with TROP2 H-scores ≥ 200 experienced markedly reduced OS compared to those with scores < 200 (*p* = 0.00021).

### Disease-free survival (DFS)

TROP2 expression metrics were also strongly associated with disease-free survival:

#### TROP2 intensity

- patients exhibiting strong TROP2 intensity (3+) had significantly shorter DFS compared to those with moderate and weak intensity (2 + and 1+, respectively) (p **< 0.001)**.

#### H-Score

- Elevated TROP2 H-scores (≥ 200) were independently linked to significantly shorter DFS (p **< 0.001**).

Multivariate analysis identified TROP2 intensity, TROP2% expression, and TROP2 H score as independent predictors of overall survival (OS) with statistical significance (*P* = 0.02, 0.001, and 0.012, respectively). Additionally, these parameters were determined to be independent prognostic factors for disease-free survival (DFS), with corresponding p-values of 0.002, 0.009, and 0.002.

### Regression analysis

Regression analysis identified TROP2 expression as an independent prognostic factor for survival outcomes. Specifically:

- TROP2 intensity scores of 2 + and 3 + were significantly associated with shorter overall survival (OS) (*p* = 0.003).
- The extent of TROP2 expression also emerged as an independent predictor of reduced OS (*p* = 0.002).
- TROP2 H-scores, with values ranging from 100 to 300 considered as one group, demonstrated a statistically significant correlation with shorter OS (*p* = 0.007).
- Regarding disease-free survival (DFS):
- TROP2 H-scores ≥ 100 were independently associated with shorter DFS (*p* = 0.002).
- TROP2 intensity was also a significant predictor of reduced DFS (*p* = 0.002).
- Similarly, an increased extent of TROP2 expression was linked to a higher likelihood of recurrence (*p* = 0.006).

These findings underscore the prognostic value of TROP2 expression metrics for both OS and DFS.

**Table 1:** Distribution of clinical variables among the study cohort (*N* = 80)

Clinical Variable	Category	Frequency (*n*)	Percentage (%)
Age (years)	< 53.5	39	48.80%
	> 53.5	41	51.20%
Laterality	Right	38	47.50%
	Left	40	50.00%
	Bilateral	2	2.50%
Tumor Site	Upper Outer Quadrant (UOQ)	50	62.50%
	Upper Inner Quadrant (UIQ)	2	2.50%
	Lower Outer Quadrant (LOQ)	4	5.00%
	Lower Inner Quadrant (LIQ)	14	17.50%
	Retroareolar Region	10	12.50%
Type of Surgery	Conservative Breast Surgery (CBS)	49	61.20%
	Modified Radical Mastectomy (MRM)	31	38.80%

**Table 2:** Clinicopathologic factors in relation to TROP2 expression

					TROP2 Intensity				TROP2% Expression			TROP2 H score			
**Clinicopathologic**				**Total**	**1+**	**2+**	**3+**	***P value***	**≤ 50%**	**> 50%**	***P value***	**< 100**	**100–199**	**200–300**	***P value***
					**11** **(14%)**	**36 (45%)**	**33** **(41%)**		**19** **(24%)**	**61** **(78%)**		**18** **(23%)**	**37** **(46%)**	**25** **(31%)**	
Tumor Size		< 3 cm			13 (72%)	15 (41%)	34	**0.00652**	11 (58%)	23 (38%)	0.12	13 (72%)	15 (41%)	6 (24%)	**0.00652**
		≥ 3 cm			5 (28%)	22 (59%)	46		8 (42%)	38 (62%)		5 (28%)	22 (59%)	19 (76%)	
Tumor Type		IDC		68	10 (91%)	27 (75%)	31 (94%)	0.078	15 (79%)	53 (87%)	0.47	17 (94%)	28 (76%)	23 ’ (92%)	**0.14**
		Other types		12	1 (9.1%)	9 (25%)	2 (6.1%)		4 (21%)	8 (13%)		1 (5.6%)	9 (24%)	2 (8%)	
DCIS		Absent		62	6 (55%)	29 (81%)	27 (82%)	0.21	14 (74%)	48 (79%)	0.75	13 (72%)	26 (70%)	23 (92%)	**0.089**
		Present		18	5 (45%)	7 (19%)	6 (18%)	0.21	5 (26%)	13 (21%)		5 (28%)	11 (30%)	2 (8%)	
Nodal Status		Negative		38	10 (91%)	18 (50%)	10 (30%)	**0.00212**	16 (84%)	22 (36%)	0.000243	14 (78%)	15 (41%)	9 (36%)	**0.013**
		Positive		42	1 (9.1%)	18 (50%)	23 (70%)		3 (16%)	39 (64%)		4 (22%)	22 (59%)	16 (64%)	
Capsular invasion		Present		17	0 (0%)	7 (39%)	10 (43%)	1	0 (0%)	17 (44%)	0.26	1 (25%)	10 (45%)	6 (38%)	**0.73**
		Absent		25	1 (100%)	11 (61%)	13 (57%)		3 (100%)	22 (56%)		3 (75%)	12 (55%)	10 (62%)	
Lympho-vascular emboli		Absent		30	7 (64%)	13 (36%)	10 (30%)	0.16	13 (68%)	17 (28%)	**0.00143**	11 (61%)	12 (32%)	7 (28%)	**0.059**
		Present		50	4 (36%)	23 (64%)	23 (70%)		6 (32%)	44 (72%)		7 (39%)	25 (68%)	18 (72%)	
TNM stage		I		10	10 (91%)	0 (0%)	1 (2.8%)	**< 0.001**	6 (32%)	4 (6.6%)	**0.013**	9 (50%)	1 (2.7%)	0 (0%)	**< 0.001**
		II		64	1 (9.1%)	35 (97%)	28 (85%)		13 (68%)	51 (84%)		9 (50%)	35 (95%)	20 (80%)	
		III		6	0 (0%)	1 (2.8%)	5 (15%)		0 (0%)	6 (9.8%)		0 (0%)	1 (2.7%)	5 (20%)	
T Stage		T1		18	11 (100%)	7 (19%)	0 (0%)	0.00427	8 (42%)	10 (16%)	0.075	11 (61%)	7 (19%)	0 (0%)	**,0.001**
		T2		50	0 (0%)	26 (72%)	24 (73%)		8 (42%)	42 (69%)		6 (33%)	26 (70%)	18 (72%)	
		T3		10	0 (0%)	3 (8.3%)	7 (21%)		3 (16%)	7 (11%)		1 (5.6%)	4 (11%)	5 (20%)	
		T4		2	0 (0%)	0 (0%)	2 (6.1%)		0 (0%)	2 (3.3%)		0 (0%)	0 (0%)	2 (8%)	
LN stage		N0		38	10 (91%)	18 (50%)	10 (30%)	**0.004**	16 (84%)	22 (36%)	0.00101	14 (78%)	15 (41%)	9 (36%)	**0.0103**
		N1		39	1 (9.1%)	18 (50%)	20 (61%)		3 (16%)	36 (59%)		4 (22%)	22 (59%)	13 (52%)	
		N2		2	0 (0%)	0 (0%)	2 (6.1%)		0 (0%)	2 (3.3%)		0 (0%)	0 (0%)	2 (8%)	
		N3		1	0 (0%)	0 (0%)	1 (3%)		0 (0%)	1 (1.6%)		0 (0%)	0 (0%)	1 (4%)	
Recurrence		No		59	11 (100%)	36 (100%)	12 (36%)	**< 0.001**	16 (84%)	43 (70%)	0.37	18 (100%)	32 (86%)	9 (36%)	**< 0.001**
		Yes		21	0 (0%)	0 (0%)	21 (64%)		3 (16%)	18 (30%)		0 (0%)	5 (14%)	16 (64%)	
Local Recurrence		No		75	11 (100%)	36 (100%)	28 (85%)	**0.0266**	19 (100%)	56 (92%)	0.33	18 (100%)	37 (100%)	20 (80%)	**0.00267**
		Yes		5	0 (0%)	0 (0%)	5 (15%)		0 (0%)	5 (8.2%)		0 (0%)	0 (0%)	5 (20%)	
Metastatic Recurrence		Absent		63	11 (100%)	36 (100%)	16 (48%)	**< 0.001**	16 (84%)	47 (77%)	0.75	18 (100%)	32 (86%)	13 (52%)	**0.000230**
		Present		17	0 (0%)	0 (0%)	17 (52%)		3 (16%)	14 (23%)		0 (0%)	5 (14%)	12 (48%)	
Death		No		65	11 (100%)	36 (100%)	18 (55%)	**< 0.001**	17 (89%)	48 (79%)	0.5	18 (100%)	33 (89%)	14 (56%)	**0.00021**
		Yes		15	0 (0%)	0 (0%)	15 (45%)		2 (11%)	13 (21%)		0 (0%)	4 (11%)	11 (44%)	

**Table 3:** TNBC molecular subtypes in relation to TROP-2 expression

TROP-2 Parameter	Category	BL (*n* = 40)	LAR (*n* = 8)	M (*n* = 32)	Total (*n* = 80)	*p*-value
TROP-2% Expression	≤ 50%	5	5	9	19	**0.008**
	> 50%	35	3	23	61	
TROP-2 Intensity	1+	1	5	5	11	**< 0.001**
	2+	6	3	27	36	
	3+	33	0	0	33	
TROP-2 H-Score	< 100	2	8	8	18	**< 0.001**
	100–199	14	0	23	37	
	200–300	24	0	1	25	

**Table 4:** Distribution of treatment modalities in the study cohort (*n* = 80)

Treatment Modality	Subcategory	*n* (%)
Chemotherapy	Adjuvant	71 (88.8%)
	Neoadjuvant	2 (2.5%)
	Both	7 (8.8%)
Radiotherapy	Adjuvant	54 (67.5%)
	Palliative	4 (5.0%)
	Both	10 (12.5%)
	Not received	12 (15.0%)

## Discussion

Triple-negative breast cancer represents one of the most formidable subtypes of breast malignancies, characterized by its marked biological diversity, rapid proliferation, early metastatic spread, and the absence of actionable hormonal or HER2 targets [2, 3]. These factors collectively hinder effective treatment and underscore the pressing need for novel therapeutic avenues. One such promising molecular candidate is Trophoblast Cell Surface Antigen 2 (TROP-2), whose aberrant expression has provided important insights into TNBC biology and enabled the development of targeted interventions [4, 9].

In this study, we systematically assessed TROP-2 expression using a combination of intensity scoring, percentage of stained tumor cells, and composite H-scores. Our findings revealed that elevated TROP-2 levels were significantly linked to poorer prognostic outcomes, including reduced overall survival (OS) and disease-free survival (DFS). These observations align with those reported by Ambrogi et al. and Izci et al., who similarly identified high TROP-2 expression as a marker of unfavorable prognosis in TNBC cohorts [18, 37].

Moreover, TROP-2 overexpression in our dataset was associated with clinicopathologic indicators of tumor aggressiveness, notably increased tumor size and nodal metastases. These associations reinforce TROP-2’s role in cancer progression, consistent with mechanistic evidence from Li et al. and Guan et al., who showed that TROP-2 activates pro-oncogenic pathways—such as PI3K/AKT and ERK/JNK—thereby promoting cell proliferation, migration, and epithelial-mesenchymal transition (EMT), a hallmark of metastasis and therapeutic resistance [15, 17].

Our analysis also explored TROP-2 expression across molecular subtypes of TNBC. We observed that the basal-like subtype demonstrated significantly higher TROP-2 expression compared to the luminal androgen receptor (LAR) and mesenchymal (M) subtypes. These subtype-specific differences highlight the potential of TROP-2 as a biomarker for molecular stratification and personalized therapy.

Additionally, the study revealed that high TROP-2 expression may serve as a predictor of suboptimal response to systemic therapies. Patients with strong TROP-2 staining exhibited lower survival following neoadjuvant or adjuvant chemotherapy, potentially due to activation of survival pathways or modulation of drug transport mechanisms. Previous work by Liu et al. and Spring et al. supports this association, implicating TROP-2 in therapy resistance via EMT-related pathways that limit drug sensitivity [42.43]. These findings underscore the utility of TROP-2 not only as a prognostic marker but also as a guide for predicting chemoresponsiveness.

From a therapeutic standpoint, targeting TROP-2 has gained momentum with the development of sacituzumab govitecan, an antibody-drug conjugate (ADC) designed to selectively deliver the cytotoxic agent SN-38 to TROP-2–expressing cells. Clinical evidence, including the pivotal ASCENT trial, has demonstrated meaningful improvements in both progression-free and overall survival among patients with refractory TNBC receiving this agent [7, 41, 42].

Despite these advances, therapeutic resistance and heterogeneity remain key obstacles. Variable TROP-2 expression within tumors may compromise treatment efficacy, while resistance may arise through downstream signaling alterations or drug efflux adaptations [20]. These complexities advocate for combination strategies, potentially incorporating TROP-2-targeted therapies with immune checkpoint inhibitors or PARP inhibitors, to augment response and mitigate resistance [14].

Notably, the involvement of TROP-2 in epithelial-to-mesenchymal transition and its expression on circulating tumor cells (CTCs) adds further relevance. Yu et al. demonstrated that TROP-2-positive CTCs contribute to immune escape and distant metastases, reinforcing its potential as both a systemic prognostic marker and a therapeutic target in metastatic TNBC [48].

In summary, our findings affirm that TROP-2 expression holds substantial prognostic and predictive value in TNBC. Its correlation with adverse clinical outcomes and aggressive pathological features underscores its importance in disease stratification and management. Incorporating TROP-2 assessment into clinical workflows could enhance personalized treatment planning, especially as newer generations of ADCs and combination regimens are developed to address heterogeneity and resistance in this difficult-to-treat cancer subtype.

## Conclusion

TROP-2 serves as a critical prognostic marker and a promising therapeutic target in triple-negative breast cancer (TNBC). Elevated TROP-2 expression correlates with poor clinical outcomes, aggressive disease features, and resistance to treatments. Targeted therapies, such as sacituzumab govitecan, offer potential advances in TNBC treatment, though challenges like intratumoral heterogeneity and resistance remain. Future research should focus on improving TROP-2 targeting and exploring combination therapies to optimize treatment efficacy.

## Data Availability

The data and materials are available from the corresponding author upon reasonable request.
